# Supplementary material for: Causal Relationship between Aging and Anorexia Nervosa: A White-Matter-Microstructure-Mediated Mendelian Randomization Analysis
Source: Biomedicines. 2024 Aug 16;12(8):1874. doi: 10.3390/biomedicines12081874 (PMC11351342; doi:10.3390/biomedicines12081874)
Supplement: Supplementary file 1 [file biomedicines-12-01874-s001.zip › Supplementary Figure/Figure S4/Singe SNP plot of MR analysis for TL to ubm-b-1481.pdf]

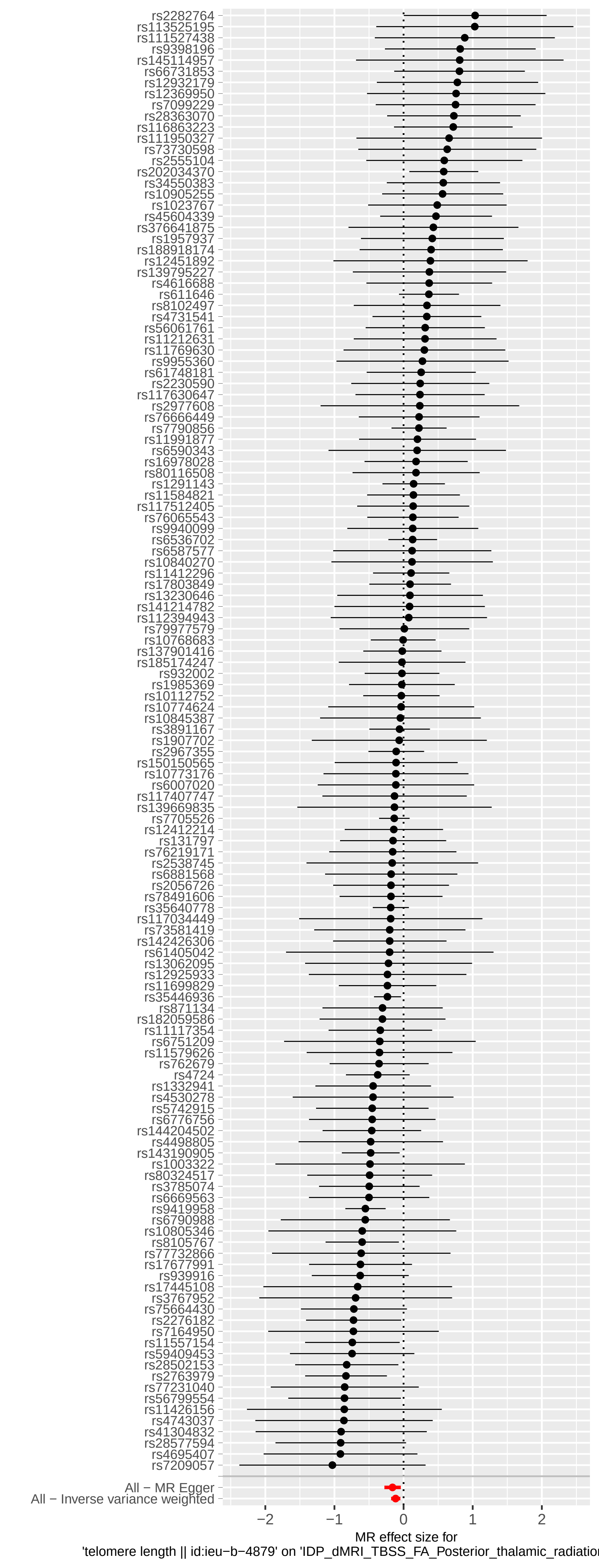
rs2282764
rs113525195
rs111527438
rs9398196
rs145114957
rs66731853
rs12932179
rs12369950
rs7099229
rs28363070
rs116863223
rs111950327
rs73730598
rs2555104
rs202034370
rs34550383
rs10905255
rs1023767
rs45604339
rs376641875
rs1957937
rs188918174
rs12451892
rs139795227
rs4616688
rs611646
rs8102497
rs4731541
rs56061761
rs11212631
rs11769630
rs9955360
rs61748181
rs2230590
rs117630647
rs2977608
rs76666449
rs7790856
rs11991877
rs6590343
rs16978028
rs80116508
rs1291143
rs11584821
rs117512405
rs76065543
rs9940099
rs6536702
rs6587577
rs10840270
rs11412296
rs17803849
rs13230646
rs141214782
rs112394943
rs79977579
rs10768683
rs137901416
rs185174247
rs932002
rs1985369
rs10112752
rs10774624
rs10845387
rs3891167
rs1907702
rs2967355
rs150150565
rs10773176
rs6007020
rs117407747
rs139669835
rs7705526
rs12412214
rs131797
rs76219171
rs2538745
rs6881568
rs2056726
rs78491606
rs35640778
rs117034449
rs73581419
rs142426306
rs61405042
rs13062095
rs12925933
rs11699829
rs35446936
rs871134
rs182059586
rs11117354
rs6751209
rs11579626
rs762679
rs4724
rs1332941
rs4530278
rs5742915
rs6776756
rs144204502
rs4498805
rs143190905
rs1003322
rs80324517
rs3785074
rs6669563
rs9419958
rs6790988
rs10805346
rs8105767
rs77732866
rs17677991
rs939916
rs17445108
rs3767952
rs75664430
rs2276182
rs7164950
rs11557154
rs59409453
rs28502153
rs2763979
rs77231040
rs56799554
rs11426156
rs4743037
rs41304832
rs28577594
rs4695407
rs7209057
All − MR Egger
All − Inverse variance weighted
−2
−1
0
1
2
MR effect size for
'telomere length || id:ieu−b−4879' on 'IDP_dMRI_TBSS_FA_Posterior_thalamic_radiation
